# Trends in Health Behavior of Polish Women in 1986–2021: The Importance of Socioeconomic Status

**DOI:** 10.3390/ijerph20053964

**Published:** 2023-02-23

**Authors:** Monika Lopuszanska-Dawid

**Affiliations:** Department of Human Biology, Faculty of Physical Education, Józef Piłsudski University of Physical Education in Warsaw, Marymoncka 34, 00-968 Warsaw, Poland; monika.lopuszanska@awf.edu.pl; Tel.: +48-22-834-04-31

**Keywords:** physical activity, alcohol, smoking, coffee drinking, SARS-CoV-2, pandemic, COVID-19, stress, Poland

## Abstract

In the last 35 years Poland has undergone a series of fundamental economic, social, and biological transformations. With the transition from a centrally planned to a free-market economy, a period of economic and social transformation, Poland’s accession to the European Union, and the COVID-19 coronavirus pandemic, living conditions in the country have seen dramatic changes. The aim of this study was to assess whether there were changes in the basic health behaviors of Polish women, and if so, in what directions and with what strength, and whether there were differences in these changes depending on the socioeconomic status. Information on basic lifestyle factors (drinking alcohol, smoking, coffee drinking, and physical activity) and socioeconomic status (level of education, Gini coefficient, Gender Inequality Index, women total employment, employed women being in managerial positions, women among scientists) of 5806 women aged 40–50 years were analyzed. During the 1986–2021 period, based on the same methodology, team of technicians and research tools, six birth cohorts of women were examined in 1986, 1991, 1996, 2006, 2019 and 2021. Highly statistically significant changes were found in the frequencies of declared health behaviors from 1986–2021, according to the order of significance in coffee and alcohol consumption, physical activity levels, and smoking and smoking intensity. In subsequent cohorts, there were fewer and fewer women who did not drink coffee and alcohol, while more drank more than two cups of coffee a day and drank alcohol more often than 2× a week. Furthermore, they were more likely to be physically active, and slightly fewer were smokers. The lifestyles of the women were less likely to depend on their socio-economic status than the cohorts. In 1991 and 1996, there was a marked intensification of unhealthy behavior. Changes in the analyzed health behaviors may have been caused by adaptation to the high level of psychosocial stress observed during the transition of the 1986–2021 period and may result in changes in the biological condition and quality and length of life of Polish women. Research on social differences in health behavior provides an opportunity to analyze the biological effects of changes in the living environment.

## 1. Introduction

In the last 35 years, Poland, like other countries in Central and Eastern Europe, has undergone a series of fundamental economic, social, and biological transformations. They started with the massive economic and social transformation from a centrally-planned economy typical of communist countries to a free-market economy, followed by Poland’s accession to the European Union, and the COVID-19 coronavirus pandemic. During this period, the polish population was subjected to a kind of unique and unplanned biological and historical “experiment”. Both phenomena have been associated with fundamental social changes, leading to dramatic changes in everyday life and significant economic consequences. These phenomena have brought a heightened sense of chronic insecurity about the health and lives of most people in Poland. In the last years of the period studied, there has been an unprecedented phenomenon with a significant impact on daily life: the worldwide COVID-19 pandemic associated with an increased sense of threat to health and life and social isolation.

As indicated by the findings of various studies, an unstable living environment (e.g., the social and systematic transformation of the late 1980s and early 1990s, the pandemic period) is a strong factor in psychosocial stress and a challenge that different social groups have tried to meet in different ways [1,2,3,4,5]. Numerous studies have demonstrated that broadly understood mental comfort has a direct effect on life expectancy, and the discomfort significantly increases the risk of premature death [6,7,8]. Changes in the broadly understood living environment of the Polish population had a clear impact on the lifestyles of Poles and on their health status. It is proven that high levels of stress can contribute to a number of unhealthy behaviours and habits, e.g., excessive consumption of high-calorie foods, abuse of alcohol and medicines, smoking, drug use, deterioration of sleep quality, avoidance of physical activity or general apathy [9,10]; although results in this regard remain partial and inconsistent. Diversified skills in coping effectively with stressful situations and accumulating the negative effects of chronic stress can lead to health differences between groups or social strata. With the differentiated ability to adapt to the new reality, both of individuals and entire social groups, the effects of psychosocial stress of the period 1986–2021 could make the biological effects of perceived psychosocial stress temporally and socially differentiated, thus perhaps manifesting gradients in lifestyle and biological condition [11].

Studies have also demonstrated that many biological features of the body are highly dependent on the position of the individual on the scale of social prestige, e.g., educational level or social position [4,12,13]. The lower the position of individuals on the educational scale, the worse the broadly defined biological condition. Studies of the Polish population have shown that people with lower education, for example, have less favorable biological parameters of bone mineralization [14], lower body height [4,11], are more obese [11,15], age faster [16,17,18], and have higher mortality rates [19].

The significant biological differences between the representatives of different education categories are mainly associated with different levels of health awareness, and different lifestyles and models of healthy and unhealthy behavior [20,21]. Furthermore, there are temporal secular trends in human biological traits that reflect changes in the living conditions of given birth cohorts [4,11,19].

Living conditions over the past 35 years have been fraught with stressors that may have affected women’s lifestyles. Therefore, it is undoubtedly interesting to attempt to assess changes over time in the basic elements of the lifestyle of Polish women and to determine which basic factors, including time (successive years of the study, cohorts) or educational level (being a good measure of social standing) are more responsible for the lifestyle. Assessing lifestyle changes, their strength and direction, and learning about the strength of the impact of the analyzed factors on women’s lifestyle can be of great practical importance, mainly by identifying groups at risk of deterioration of the biological condition and shorter healthy life expectancy, i.e., groups with lower capacity to adapt to a changing environment.

## 2. Materials and Methods

### 2.1. Material

The material for the analysis consisted of data on 5806 adult Polish women aged 40–50 years of the Caucasian race, living in Polish cities with over 500,000 inhabitants. Research data of the participants were presented by year of examination: 1: 1986, 2: 1991, 3: 1996, 4: 2006, 5: 2019 and 6: 2021. The numbers of women in subsequent groups are respectively: 1: n = 2016; 2: n = 1181; 3: n = 1203; 4: n = 642; 5: n = 498; 6: n = 266, details are given in Table 1 (the number of respondents in each year of the study in accordance with the Power test at a minimum level of 0.8). The choice of women in the fifth decade of life is dictated by the fact that people in this age group are usually characterized, on the one hand, by a kind of stability in life in professional, economic and private terms, and on the other hand, it is a period of relatively rapid changes in body systems associated with entering menopause. In addition, this is an exceptionally sensitive decade in which cardiovascular diseases frequently manifest themselves. Moreover, studies of middle-aged women can provide knowledge about factors which impact longevity [22,23].

The data were collected within the framework of several research projects (1986–2021 years) financed from various non-statutory grant funds (e.g., KBN—Committee for Scientific Research, MNiSW-Ministry of Science and Higher Education grants, MEiN—Ministry of Education and Science). Research in the following years (1986, 1991, …) was carried out as part of research projects that varied thematically. The scientific goals of these projects were not focused on health behavior analysis. For the purposes of this study, the same basic questions and answers were selected from the raw data for each project. Thus, based on scientific projects carried out in a certain time sequence in the past, while maintaining consistency in research questions, it is possible to analyze trends in changes in health behaviors over 35 years. The years assigned to each cohort (1986, 1991, etc.) represent the middle year of each research project.

The average number of people participating in Polish examinations, is about 25% [6,16]. The participants included in the analysis were not selected in any way in terms of any parameters. The study group was ethnically homogeneous, without linguistic or cultural minorities. The women participating in the study did not suffer from severe chronic diseases at the time of the study or in the past. No pathologies were also found in the women in the physical examination. About 1% of the questionnaires were rejected from the final database due to the incompleteness of the answers provided to all questions. Interventionary studies involving animals or humans, and other studies that require ethical approval, must list the authority that provided approval and the corresponding ethical approval code.

### Ethical Approval

All procedures performed in studies involving human participants were in accordance with the ethical standards of the institutional and/or national research committee and with the 1964 Helsinki declaration and its later amendments or comparable ethical standards. All persons were orally informed about the aims of the projects and all testing procedures and all gave their informed consent prior to their inclusion in the study. At any time, the subjects could withdraw without giving any reason.

The grant applications to the KBN and the MNiSW were reviewed and approved by a board of experts in many fields (including ethical standards). Before the study began the board took into account the observance of ethical standards in this human research. Only projects that received the highest criteria for substantive and ethical evaluation received funding. The most recent studies have been approved of the Senate Ethics Committee for Scientific Research of the Józef Piłsudski University of Physical Education in Warsaw (SKE 01-5/2020, 20 November 2020).

### 2.2. Methods

#### 2.2.1. Collecting Data

The research was based on the same methodology, team of technicians and research tools. Surveys were conducted at each time point using a supervised survey method (paper and pencil). The participants completed questionnaires on health behavior independently in the presence of a person providing clarification, if necessary. The face-to-face interview method was not used and significantly reduced the risk of manipulating the data, for example, to gain the social approval of the interviewer [24]. Any ambiguity in the interpretation of the questions was clarified by interviewers trained for the purpose.

Survey data on basic socioeconomic status included information on the age (in years) and education of the women surveyed (data collected directly from respondents). In Poland, the educational level is still an excellent measure of the position on the social scale [4,11]. Three categories of the educational level of the women surveyed were used: (1) tertiary education, (2) secondary education, and (3) vocational education and lower. In addition, in order to assess the social and economic status of women, which well describes the situation of women on the labor market and in the economy in Poland in specific years, objective and key indicators characterizing the position of women in Polish society at a specific time were included in the analysis. The main indicators included were: Gini coefficient, Gender Inequality Index, women total employment (in %), employed women being in managerial positions (in %), women among scientists (in %) (data extracted indirectly from relevant international databases) [25].

The research questionnaire included questions on selected basic lifestyle elements of the women surveyed. In all the surveys (1–6), the same short questions were asked and the participants had the same short answers to choose from. Lifestyle was assessed based on the basic elements such as alcohol consumption, cigarette smoking, and its intensity, coffee consumption, and the level of leisure-time physical activity. Based on the answers to the question about alcohol consumption, the women surveyed were divided into three categories: (1) non-drinkers (drinking exceptionally rarely or never), (2) occasional drinkers (up to 1–2 a month), (3) frequent drinkers (more than two times a month). Responses about cigarette smoking were divided into three categories: (1) never, (2) quit smoking (at least 1 year ago), (3) smoker. Furthermore, cigarette smokers also declared how many cigarettes they smoked on average per day, with three categories to choose from: (1) less than 5 cigarettes per day, (2) 5–20 cigarettes per day, and (3) more than 20 cigarettes per day. Coffee drinking frequency was assessed using three categories: (1) “I don’t drink coffee”, (2) “I drink one or two cups a day”, and (3) “I drink three or more cups a day”. The question concerning physical activity included three categories distinguished by the regularity and intensity of the declared activity: (1) physically active (regularly doing any physical activity), (2) exercising irregularly, and (3) physically inactive.

#### 2.2.2. Statistical Analysis

A non-parametric chi-squared test χ^2^ was used to assess the significance of differences in the frequencies of specific education categories and health behavior between years (cohorts), and within educationally homogeneous groups between years of the study. The level of significance was set at α = 0.05. All significance levels are given to the nearest 0.0001. Logistic regression analysis was used to assess the risk of individual unhealthy behaviors for subsequent study years and educational levels. Odds Ratio (OR), 95% confidence intervals (95%CI), McFadden’s R-squared values of determination, Hosmer-Lemeshow test for goodness of fit for logistic regression models and levels of statistical significance (*p*) have been given. The STATISTICA 12.0 and 13.5 packages were used for analyses [26].

## 3. Results

Frequencies for data collected directly from respondents: educational level, lifestyle parameters for each study year, and chi-squared test values for the significance of changes over time were presented in Table 1. Changes in the educational structure of the women surveyed during the period studied were large and highly statistically significant (χ^2^ = 856.21; *p* = 0.0001). In subsequent cohorts, there was a noticeable increase in the percentage of women with tertiary education. There was an increase between the 1986 and 2021 cohorts in the percentage of the most educated women, from 23.8% and 31.9% in 2006 to 63.5% in 2021. At the same time, the percentage of women with primary education decreased significantly, from 34.7% in 1986 to 3.7% in 2021. In general, the largest group throughout the period studied was women with secondary education (about 37.01% on average).

The variation in the intensity of drinking alcohol during the study period was highly statistically significant (χ^2^ = 1808.02; *p* = 0.0001). In all cohorts, the vast majority of women reported only occasional drinking, and the percentage of these participants ranged from 50.99% in 1986 through 78.51% in 2006 to 73.31% in 2021. At the same time, there was a very marked decline in the number of abstinent women, from ca. 42.21% in 1986 and 7.48% in 2006 to 11.28% in 2021. In contrast, the percentage of women reporting frequent drinking increased nearly 2.5-fold, from 6.80% in 1986 to 15.41% in the latest cohort, with cumulative percentages of heavy drinkers in 1991 and 1996 (respectively, 59.95% and 36.49%) (Table 1).

The change in smoking frequency in subsequent cohorts showed significant variation (χ^2^ = 354.65; *p* = 0.0001). Despite the high fractions of female smokers in each study cohort (average for surveys 1–6: 35.95%), a positive slight decrease was observed in the fraction of female smokers, especially after the 1990s. Across cohorts 1–6, the percentage of women giving up their smoking habit nearly doubled from 11.36% in 1986 by 21.03% in 2006 and 23.68% in 2021. The fraction of non-smokers ranged from 34.41% to 68.27%. The largest numbers of women declaring to smoke cigarettes, 47.47% and 41.16%, were found for the 1996 and 1991 cohorts, respectively. Furthermore, the fewest women (just 5.33%) declared quitting smoking in 1991 (Table 1).

Analysis of the intensity of cigarette smoking by female smokers indicated a worrying trend: the number of cigarettes smoked a day has clearly increased. Between 1986 and 2021, the fraction of women claiming to smoke more than five cigarettes a day increased from nearly 50% in 1986 to around 80% in almost all subsequent cohorts (except 2019, with 57%). The number of women smoking more than 20 cigarettes a day increased significantly in that period, from 14.25% in 1986, a peak of 30.24% in 1991 to about 21–26% in 1996–2019, and 15.79% in the latest survey. The most unfavorable pattern of cigarette smoking was observed in 1991, when as many as 83.74% of women smoked more than 5 cigarettes a day, of which 30.24% smoked very heavily (more than 20 cigarettes a day) (Table 1).

The analysis of changes in the amount of coffee drunk by the women studied over the period studied showed the greatest significance of differences in frequencies compared to other lifestyle elements (χ^2^ = 2439.36; *p* = 0.0001). Throughout the period, on average, about 40% of women drank 1–2 cups of coffee a day. There was a marked decrease in the percentage of women not drinking coffee, from 47.67% in 1986 to just 2.91% in 1996 and 11.27% in 2021. At the same time, the percentage of women drinking more than two cups of coffee a day has increased significantly in recent years, from 5.85% at the beginning of the period studied, 76.56% in 1996, and 18.05% in 2021, with an average of about 35.14% for the entire period 1–6 (Table 1).

The frequency distribution of the different levels of physical activity of the women surveyed changed significantly between 1986 and 2021 (χ^2^ = 1681.55; *p* = 0.0001). These findings demonstrated a low level of leisure-time physical activity of women but they also indicated a positive trend of the gradually increasing percentage of women who were physically active on regular basis (Table 1).

The percentage of women regularly involved in some forms of physical activity increased significantly, especially since the late 1990s. In the surveys conducted in 1996, 2006, 2019 and 2021, the percentage of physically active women at least doubled compared to 1986, and, in 2019, reached 69.48%. The percentage of women reporting low physical activity, passive leisure-time activities or irregular physical activity, dropped from nearly 77% in 1986 to about 57% in 2021. A disturbing phenomenon is the doubling of the number of women who spend their leisure time completely passively, rising from 15.77% in 1986 to 31.62% in 2006, with the number declining again to around 16% in the latest cohort.

Table 2 presents the frequencies of categories of individual health behaviors in successive cohorts within homogeneous educational groups (data collected directly from respondents), the significance of differences in these frequencies, and the significance of differences in the frequencies of categories of behavior regardless of the year of the study but depending on the educational level. For all lifestyle elements, statistically significant differences were found in the reported categories of behavior between educational groups (results presented in the last column of Table 2). The largest differences were observed for alcohol drinking (χ^2^ = 358.47; *p* = 0.0001), followed by physical activity (χ^2^ = 213.00; *p* = 0.0001), coffee drinking (χ^2^ = 144.14; *p* = 0.0001), cigarette smoking, and the number of cigarettes smoked (respectively χ^2^ = 110.34, *p* = 0.0001 and χ^2^ = 17.61, *p* = 0.0015). Differences in the frequencies of health behavior categories between successive surveys (1–6) were found to be significantly greater than differences between educational groups. For example, for alcohol drinking, the differences in frequencies in the 1986–2021 period in subsequent behavioral categories of tertiary or vocational education are about 2 times greater than the differences based on educational level (respectively χ^2^ = 833.44 and χ^2^ = 633.79 relative to χ^2^ = 358.47). An analogous direction of the relationship was found for all health behaviors. The largest relative differences were noted for coffee drinking, for which the importance of time (cohorts 1–6) is about 6–7 times more strongly a differentiating factor in coffee drinking habit than educational level (e.g., χ^2^ = 1073.07 for tertiary education in 1986–2021 vs. χ^2^ = 144.14 for the importance of the level of education, regardless of the year of the study). Therefore, despite statistically significant differences in individual categories of health behavior, it is the cohort that is by far the stronger differentiating factor of health habits than education (Table 2).

Table 3 illustrates the results of logistic regression analysis, indicating the odds ratio (OR) for the prevalence of negative health behaviors (unhealthy behaviors) in successive years of the study (with 1986 as the reference group) and in individual educational levels (tertiary education as the reference group) (analysis including data collected directly from respondents). The risk of heavy alcohol consumption by the women surveyed during the period studied increased markedly in 1991 (more than 18 times) (OR = 18.3546), followed by a double decline in 1991–2021 (respectively for years 2019 and 2021, OR = 1.7028, OR = 2.0486). As the educational level decreased, the risk of heavy alcohol consumption decreased.

Compared to 1986, the risk of smoking cigarettes was 32% higher in 1991 (OR = 1.3153) and about 79% higher in 1996 (OR = 1.7900). In subsequent cohorts, the risk of smoking was lower than in 1986, with the lowest OR recorded for 2019 (OR = 0.4922). As the level of education decreased, the risk of smoking increased. For women with vocational education, it was about 41% higher than for women with tertiary education (OR = 1.4056).

The risk of the intensity of smoking more than 20 cigarettes a day for women in 1991 was almost 3 times higher than in 1986 (OR = 2.6970) and about 2 times higher in 1996 and 2006 (respectively OR = 1.8437 and OR = 2.1607).

The habit of drinking more than two cups of coffee a day was about 20 times more frequent in 1991 than in 1986 (OR = 19.9733), and about 60 times more frequent in 1996 (OR = 60.3562). In the subsequent years of the study, the risk of consuming this amount of coffee decreased to about 3 times that of the baseline year (OR = 2.8215). Women with secondary and vocational education had a lower risk of consuming more than two coffees per day (respectively OR = 0.3509 and OR = 6353).

With subsequent cohorts, the risk of being physically inactive at first significantly increased almost 8-fold (1986: OR = 7.8062) and decreased in 2019 to OR = 0.25 and then in 2021, and slightly increased to the level recorded in 1986 (OR = 0.9002). As the educational level decreased, the risk of being physically inactive decreased slightly (e.g., for vocational OR = 0.7449).

The values of all χ^2^ tests, McFadden R-square coefficients of determination oscillating from 0.2858 to 0.9153, and the values of all Hosmer-Lemeshow tests for logistic analysis indicated goodness-of-fit for the analyses performed in the study (value *p* from 0.0070 to 0.0001) (Table 3).

Table 4 presents the results of a logistic analysis in which, in addition to data obtained directly from the respondents, five indicators characterizing the general situation of women in Polish society and the labor market in specific years were included in the variables determining health behavior. The inclusion of indirectly acquired variables in the logistic analysis increased the goodness-of-fit of the models in most cases, which can be seen in the higher values of the Hosmer-Lemeshow test (e.g., for smoking, the test value increased from 27.3277; 0.0001 to 61.4640; 0.0001). The exception is the effect of the determinant variables on the risk of unhealthy alcohol consumption, for which the goodness of fit of the model decreased (from 79.1956; 0.0001 to 16.0403; 0.0248). With regard to almost all of the baseline health behaviours analysed, the cohort (and therefore the importance of change over time) remained an exceptionally strong predictor of health behavior change, reaching the absolute highest OR values for the risk of unhealthy alcohol consumption, smoking, coffee consumption or the risk of being physically inactive. The second most important predictor of health behavior was found to be the indicator of employed women being in managerial positions. It is a significant factor for, in turn, drinking alcohol, smoking cigarettes and drinking coffee. As the frequency of women being in managerial positions increases, the frequency of unhealthy health behaviours increases (e.g., in the case of frequent alcohol consumption OR = 25.6732; *p* = 0.0001). The Gender Inequality Index and, further down, the Gini coefficient also reach significance (Table 4). In general, as the number of female employees and the number of female scientists increases, the risk of anti-health behavior decreases.

## 4. Discussion

An undeniably positive effect of the ongoing economic and social changes in the 35 years of 1986 to 2021, especially the beginning of the period, was the rapidly increasing access to consumer goods in the form of both domestic and imported products to meet basic needs and durable goods [27]. A number of source data indicate that access to goods and their flexible prices have caused significant changes in lifestyle patterns, often in a health-promoting direction. Noticeable directional changes were observed in basic lifestyle elements that are highly relevant to health, with different educational groups seeming to respond somewhat differently to the changing reality. In the 1990s, marked changes were noted in increased unhealthy behavior, intensifying alcohol consumption, cigarette smoking, and increased physical inactivity. These changes may have had a significant impact on changes in the biological and functional status and the quality of life of Polish women. Of fundamental importance in the health behavior of women in Poland appears to be their situation in the labor market and the economy.

### 4.1. Alcohol Drinking

A clear trend of a highly significant increase in alcohol consumption was observed during the period studied, especially the years of economic transition in Poland and other Central and Eastern European countries [21,28,29]. The annual consumption of all alcoholic beverages in Poland in units of pure alcohol per capita aged 15 years or older, declined from 9.5 L in 1988 to 7.7 L in 2001, followed by a rise to 8.8 L in 2006, and 9.8 L in 2019, 9.6 L in 2020, and 9.7 L in 2021 [30]. The fluctuation in alcohol consumption between 1986 and 2021 was accompanied by a marked change in drinking patterns. In the late 1980s and early 1990s, hard liquor (e.g., vodka) was mainly consumed, while consumption of spirits declined and consumption of lower-alcohol beverages, such as wine and beer, increased in subsequent years [31]. Between 1986 and 2021, the amount of beer consumed in Poland almost tripled (from 1.9 L to 5.2 L of pure alcohol) [31]. The amount of wine consumed between 1986 and 1995 declined from 1.9 L to 0.9 L of pure alcohol, only to rise to 1.4 L in the next five years, before dropping to 1.1 L in 2006 [31] and just 0.8 L in 2020. However, since the early 2000s, there has been a trend of successively increasing the consumption of spirits again. Between 2001 and 2021, their consumption increased from 1.7 L to 3.7 L [32]. The observed temporal trends in changes in the quantity and quality of alcohol consumed depended on access to a particular type of alcohol. The onset of the free economy in Poland and the opening of the market to foreign goods have resulted in an influx of new alcoholic stimulants that are attractive to Poles. Increased alcohol consumption in Poland immediately after the privatization of the state-owned enterprises manifested itself in increased hospitalizations for alcohol psychosis or alcohol dependence, admissions to psychiatric hospitals for alcohol-related disorders, increased mortality rates from liver cirrhosis, and increased traffic accidents due to driving under the influence of alcohol [33].

The results of a study of alcohol consumption patterns in Poland [34] showed that nearly three-quarters of women consumed alcohol. Polish women drink much less alcohol on average than men and experience related problems much less often. Men drink alcohol almost three times more often than women (average 106 days per year vs. 37 days). The difference is mainly attributable to men’s more frequent beer drinking (an average of 98 days per year for men and 21 days per year for women) and more than twice as frequent spirits drinking (20 days) as for women (9 days). In the case of wine, it is women who drink almost twice as often as men (18 days vs. 10 days). Women drink almost four times less pure alcohol per year than men (the average is 2.2 L for women, and 8.1 L for men). An increase in alcohol consumption was also noted in the women surveyed. Among the respondents, the percentage of women who did not drink alcohol was found to have decreased from 42.21% in 1986 to 11.28% in 2021, with an increase in the number of women reporting frequent drinking. This is in line with the decreasing difference in drinking between men and women found in other European countries, mainly due to an increase in women’s consumption of alcohol, probably lower-alcohol beverages (mainly wine), especially in the group aged over 40 years [35]. Similar trends have been observed in Russia, Lithuania, and Estonia [36].

### 4.2. Tobacco Smoking

Another highly unhealthy lifestyle element affecting the health of the Polish female population is smoking. In general, the women surveyed reported quitting cigarette smoking more frequently from cohort to cohort, but a particularly negative aspect of cigarette smoking by the women surveyed is that smokers, in successive cohorts, reported smoking more and more cigarettes a day. Female smokers tended to smoke 5 to 20 cigarettes over time compared to smoking fewer cigarettes. The unfavorable trends in smoking patterns among Polish women are confirmed by WHO data [30] and other surveys [11,21]. The reduction in the frequency of cigarette smoking among men and the increase in the number of men declaring quitting smoking observed between 1984 and 1999 was not reflected in women [21,37]. Women, regardless of their education, were less likely to quit smoking than men, and there was an additional increase of more than 18% of smokers among women with a lower level of education.

The phenomenon of women’s intensification of their smoking habit and women’s higher biological sensitivity to the toxic effects of cigarette smoke appears to be responsible for the increase in women’s deaths from lung and other tobacco-related cancers, and the reduction in men’s excess mortality rates compared to women [19,38,39,40]. Studies in thirty-six European countries have shown that male mortality due to lung cancer is trending downward [41]. Furthermore, mortality rates of women from the same cause continue to rise in many countries, with mortality rates due to lung cancer continuing to rise in high-risk countries in Eastern Europe (Hungary, Poland and the Czech Republic), and Northern Europe (Denmark, Iceland and Great Britain).

Research results indicate that smoking of cigarettes may be strongly associated with the acceleration of aging processes of the female reproductive system and increasing the risk of earlier menopause [23]. Chmara-Pawlińska and Szwed indicate that non-smoking women have menopause on average two years later than women who are habitual smokers [23]. Moreover, the number of cigarettes smoked every day affected the age of menopause, accelerating its occurrence in women who smoked more than five cigarettes per day. Thus, the differentiated degree of intensification of smoking by Polish women seems to have a modifying effect on the length of the female reproductive period in the last 35 years.

Social motivations for smoking cigarettes and addiction to smoking as a habit rather than nicotine addiction make women more likely to relapse, more likely to smoke irregularly and to smoke very large amounts of cigarettes under stress. The smoking habit helps reduce stress and negative emotions, to which women are quite susceptible, and the main barrier to women quitting this habit is a lack of faith and skill in successfully quitting and a fear of gaining weight. Furthermore, the associations demonstrated between certain personality traits and the likelihood to smoke cigarettes confirmed that open-minded and sociable people with high ambitions for success, e.g., professional success, are highly likely to become addicted to cigarette smoking [42]. Furthermore, a disturbing trend can be observed; despite most European women quitting smoking, the percentage of Polish female smokers is amongst the highest [31].

When considering the problems of smoking addiction, it is important to be aware that in addition to its direct effects in the form of increased mortality rates from tobacco-related cancers, it also affects the increased risk of death from cardiovascular diseases [43]. According to the Centers for Disease Control and Prevention data [44], smoking puts women’s health at greater risk than men’s. Cigarette smoking shortens lives by an average of 14.5 years in women and by 13.2 years in men. Furthermore, female smokers have twice the risk of having a heart attack and developing bowel cancer, even though they smoke less than men. Female smoking is associated with more dangerous health consequences, as estrogen exacerbates the carcinogenic effects of cigarette smoke components and accelerates the growth of pathological cells in the lungs. Female smokers, more than male smokers, are at risk of chronic respiratory diseases, chronic obstructive pulmonary disease, osteoporosis, and cervical and breast cancer. Taking oral contraceptives also increases the risk of heart attack and stroke. In general, smoking cigarettes not only shortens the length of life but also a disease-free life. The etiology of smoking addiction, which is different from that of men, and the increasing percentage of female smokers show the importance of this problem.

Negative trends in cigarette smoking by women reached their peak in the initial shock period of the transition, in which the largest number, 41.16–47.47% of women reported smoking cigarettes, with the smallest number of them (5.3%) quitting the habit. Unfavorable trends in cigarette smoking, combined with other adverse health behaviors, especially during a difficult period of rapid economic transformation in Europe, worsen the quality of life long before death, further increasing the risk of cardiovascular disease, the leading cause of mortality, morbidity, and hospitalizations in women across Europe [44,45].

### 4.3. Coffee Consumption

During the period studied, the fraction of women who did not drink coffee decreased markedly, while the percentage of women who drank more than two cups of coffee a day increased from the baseline 5.85% to 76.56% in 1996 and 18.05% at the end of the period. A study of 8821 Polish men and women conducted as part of the HAPIEE project found that heavy coffee drinkers had lower relative body weight, smaller waist circumference, lower systolic and diastolic blood pressure and triglyceride levels, and higher levels of the HDL fraction of cholesterol compared to those drinking less than one cup a day [46]. It has also been proven that high coffee consumption among women (more than two cups a day) reduces the risk of metabolic syndrome by 31% compared to women who drink at most one cup of coffee a day [47]. These researchers found metabolic syndrome in 30.2% of non-coffee drinkers and in 23.7% of those who drank more than two cups of coffee a day. More and more studies confirm the health-promoting effects of coffee on the human body, although the results are still sometimes debatable [47,48,49]. A number of studies have confirmed its beneficial effects on, for example, glucose levels, insulin metabolism [50,51], BMI and waist circumference [52], or the potentially decreased risk of cardiovascular disease and cancer, especially in women [48,53]. The exact mechanism behind the health-promoting effects of drinking coffee on the human body is subject to debate but the main role is attributed to polyphenols, antioxidants, and anti-inflammatory and anti-diabetic drugs. Coffee contains significant amounts of vitamins and minerals, such as ascorbic acid (vitamin C), B vitamins, riboflavin, niacin, folic and pantothenic acids, magnesium, potassium, manganese, fluorides, chlorogenic acid, caffeine, diterpenes, trigonelline, and melanoidins. The rich composition of biologically active substances has a protective effect on the cardiovascular system and counteracts metabolic disorders [48,49,51]. The changes in coffee consumption patterns observed among the women surveyed therefore appear to be highly health-promoting.

### 4.4. Physical Activity

Human biological status is significantly dependent on the level of physical activity, especially its recreational form. The effects of physical exercise, including involvement in competitive sports, on female health and life expectancy have long been of interest to a number of researchers [54,55]. Numerous research results, including those obtained for the Polish population, have shown a significant relationship between the level of physical activity and a number of functional indicators, such as cardiovascular fitness, lipid profile, and homocysteine concentration [56,57]. The women surveyed were not very physically active in all cohorts, but there was a positive increasing trend in the percentage of women who were regularly physically active from 23.26% in 1986 to 42.48% in 2021. Unfortunately, over the period studied, the percentage of inactive women multiplied in several cohorts, from 15.77% at baseline to a dramatic 62.15% in 1991 and 16.54% in 2021. As indicated by the results of a survey of the physical activity of Poles conducted by CBOS [58], despite the fact that men and women appreciate the importance of physical exercise and recreational physical activity and being active outdoors, as many as 39% declared that they had rested passively and did not practice sports or perform any exercises for physical fitness. However, there has been a positive trend of a slow decrease over time in the number of people who were completely inactive from 74% in 1997 to 59% in 2003 and 39% in 2018 [59,60].

Other results from studies of the Polish population highlight clear social gradients in the level of recreational physical activity [21,58,60]. As the educational level decreased, the tendency toward inactive leisure activities increased. The CBOS results [58] show that among the physically active respondents, there are twice as many people with tertiary education compared to those least educated (83% vs. 42%). In a study conducted between 1984 and 1999, an increase in the frequency of regular physical activity was noted only among women with tertiary education [21]. The increase in the percentage of women declaring active leisure activities, especially women in their 40s and 50s, is likely due to increased health consciousness and also better care of their appearance. In light of the strong links between physical activity levels and perceived emotional states such as anxiety or depression proven in the literature, the trend of an increasing percentage of women who are active is favorable from the standpoint of the mental status of adult women [61].

The changes in basic health behaviors observed in the Polish population over the analyzed period were clearly reflected in healthy changes in the values of the basic somatic physique characteristics of the women studied, i.e., an increase in average body height, a decrease in relative body weight and body fat percentage, or decreasing body circumference, healthier measures of cardiovascular fitness, and biochemical blood results [4,11]. Changes in women’s health and dietary habits may also have reduced the incidence of diet-related conditions, i.e., obesity, hyperglycemia, hypercholesterolemia, and contributed to the stabilization of systolic and diastolic blood pressure and heart rate [4,11].

### 4.5. Psychosocial Stress and the Importance of Socioeconomic Status

With the generally positive trends in changes in health behavior, the sharp changes observed during the first shock years of the systemic transformation in Poland are noteworthy. High levels of psychosocial stress can induce a range of unhealthy behaviors and habits [9,10]. Different skills in coping effectively with stressful situations and accumulating the negative effects of chronic stress can lead to health differences between groups or social strata [8,16,62]. The social changes in the period studied (1986–2021), especially its first stage, related to the transition from a centrally-planned to a free market economy, proved to be so dramatic that they were associated with a great and sudden stress experienced by the entire society, including women aged 40 to 50 years. Both the direct and indirect effects of intense stress, through constant stimulation of the hypothalamic-pituitary-adrenal axis and the development of a stressful lifestyle, resulted in dramatic adverse health-related changes in the women studied. Not surprisingly, many parameters describing the biological status of the women surveyed showed their breakdown during this period [11,63]. The level of psychosocial stress experienced and differences in the level of knowledge and health awareness regarding the importance of a healthy lifestyle or the need for regular preventive examinations are considered among the key reasons for the social gradients in biological measures of health status. Some researchers have demonstrated associations between educational levels and selected lifestyle elements, including the frequency of harmful smoking habits, alcohol abuse, or levels of involvement in recreational physical activity [21,64,65]. It is common that the prevalence of risky unhealthy behaviors, such as cigarette smoking, alcohol abuse, and the prevalence of sedentary lifestyles, increase as the educational level decreases. These relationships tend to be particularly pronounced in men [66], while among women, the association of educational level with the self-reported frequency of healthy and unhealthy behaviors are not so straightforward. An example of this is the increasing percentage of women with secondary education who regularly and heavily smoke cigarettes and consume alcohol observed over the past two decades in this and previous studies [21]. More frequent use of cigarettes by these women may be a mechanism to reduce the high levels of stress resulting from the need to find the balance between meeting high demands at work, professional ambitions, and the demands of family life, and controlling appetite and protecting against unwanted weight gain [67,68]. Some studies have shown the educational level to be the most important socioeconomic factor, associated with almost all parameters of biological condition, and the significant health risks throughout the study period. Among Polish women, regular social gradients were found in most parameters of biological fitness. The different level of sensitivity of different social groups to the same external stimuli in the form of social and political changes in Poland has been confirmed by other analyses of the Polish population, such as the study of secular trends in the body height of children and adolescents or the maturation of girls [69,70]. They have indicated that certain social subgroups, such as those at the top of the social ladder, experience more dynamic changes in body height compared to lower subgroups. Therefore, the magnitude of the changes associated with Poland’s political transformation at the turn of the 21st century or the pandemic crisis may clearly differ from one social group to another.

### 4.6. COVID-19

Highly stressful situations, such as the shock period of socioeconomic transition or the COVID-19 pandemic, have negatively affected well-being, mental health, and lifestyles [71,72]. Stress is conducive to the consumption of more alcohol [73,74], increased nicotine addiction [75], snacking between meals [76], and disrupted sleep patterns [77], which can result in weight changes [78,79], increased risk of cardiovascular disease, and premature deaths. Research results in this field are still inconclusive. Some researchers point to trends in improved healthy behaviors during the pandemic, such as an increase in the number of people quitting nicotine addiction [80] or a reduction in alcohol consumption [81].

Lockdowns announced in many countries and quarantines due to COVID-19 have significantly affected the level of physical activity, inducing a change to a more sedentary lifestyle [82]. Due to the social isolation and the extensive restrictions imposed by national governments (e.g., closure of sports facilities, prohibition of access to forests in Poland), it has been quite a challenge to meet international recommendations for levels of physical activity. With the closure of sports facilities and the inability to perform their favorite physical activities, such as team games, gym workouts, and swimming, some of the respondents began to train at home and in outdoor settings [83] in an attempt to stay motivated to practice sports [84]. Unfortunately, significant decreases in physical activity have been recorded in most countries [85], which was also confirmed in the present study. In Polish women, the percentage of physically inactive respondents in 2021 increased by as much as about 400% compared to 2019 (from 5.42% in 2019 to 16.54% in 2021), while the fraction of those regularly active decreased from 69.48% to 42.48%.

### 4.7. Social Situation of Polish Women

Poland still remains a country with uneven social development of individual population groups, a particular example of which are social differences based on gender. The situation of women in the labor market and in Polish social life has improved slightly in recent years. According to the UNDP Reports on Human Development (2022) [25,86] the Gender Inequality Index in Poland (GII—a composite measure of gender inequality in three dimensions: reproductive health, empowerment and the labor market, a low GII value indicates low inequalities between women and men) since the early 1990s is successively decreasing (1991: 0.278; 1996: 0.217; 2006: 0.159; 2019: 0.111; 2021: 0.109). However, women still face difficulties in advancing in their careers and obtaining salaries commensurate with their qualifications. Gender inequalities in the labor market still exist, making it difficult for women to achieve professional success. Positive trends are also observed in other indicators, e.g., employment of women in high positions or in the parliament (from 12.7% in 1991 to 27.5% in 2021). However, this is still not enough to ensure gender equality in this area. Moreover, the percentage of women working in science in Poland has gradually increased since the 1980s. Nevertheless, there are still some challenges related to gender inequality in science, such as a lower representation of women in senior scientific positions, fewer women receiving scientific awards and less funding for research conducted by women. According to Eurostat data, in 2020, 33.6% of women aged 25–64 in Poland had a university degree [25]. It can be seen that this percentage has been steadily increasing in recent years, suggesting that more and more women in Poland are gaining tertiary education. In 2010, the percentage was 21.1% and in 2015 it was 26.7%. Nevertheless, the values of these indicators are still less favorable than the average for the European Union [86]. The improving socio-economic situation of women, increases the comfort of social functioning, but also imposes high psychological and physical costs. The direct physical cost of the high pace of life, professional, financial and family success in women is a change in basic health behavior towards masculinization. By adopting a lifestyle typical of men, with a high incidence of hazardous behavior, women reduce their psychosocial stress levels [25,87].

### 4.8. Limitation Study

The collected research material is large, but it does not constitute a strictly random sample of the female population of big cities, as it only contains information about people who volunteered and wanted to participate in the implemented grant projects. Therefore, it can be expected that if the answers were given by randomly selected women, the results would be less positive. In addition, a minor drawback of the research material may be the inequality in the number of samples from subsequent years of research (although each sample meets the requirements of the minimum sample size for the analysis). Furthermore, it would be advisable to extend the catalog of socio-economic factors with other factors, for example, professional or family situation, which would probably significantly enrich the obtained results. Another limitation of this study may be the differences between the cohorts in the relationship between the level of knowledge, skills, competences or awareness and the level of education.

## 5. Conclusions

The observed directional changes in the basic components of the lifestyle of Polish women between 1986 and 2021, resulting from changes in environmental conditions, including the level of psychosocial stress, led to variations in the biological status of Polish women [11]. The masculinization of women’s lifestyles observed in Poland, similar to the urban areas of developed countries, resulting from the increasing occupation of high-ranking positions at work and the need to balance work and family life, is associated with a high psychophysical costs. Competition at work, prolonged stress, the fast pace of life, and aspirations to achieve professional, financial, and family success are offset by changes in women’s lifestyles, which in turn are reflected in the change in the structure of causes of death over the past quarter century [11,87,88].

The behavioral implications of the intense political, economic, social, and biological changes between 1986 and 2021 indicate a slight improvement in the lifestyles of economically active women in the fifth decade of life. In addition to individual relevance, information on trends in changes in women’s basic health behaviors should have a significant impact on national health policies and the design of appropriate educational models to promote healthy lifestyles among women. New prevention programs should take into account both the vulnerability and the varying adaptability of different social groups to changes in environmental conditions. In light of the increasing life expectancy, the results obtained in the present study allow a conservative prediction for extending the number of years without chronic diseases and increasing the quality of life of adult women.

## Figures and Tables

**Table 1:** Characteristics of educational level and women’s life style in particular years (N, percentages—%) and the importance of the differences over time (Chi-square test; *p*—the level of significance).

	1: 1986		2: 1991		3: 1996		4: 2006		5: 2019		6: 2021		1–6: 1986–2021		
	n = 2016		n = 1181		n = 1203		n = 642		n = 498		n = 266		N = 5608		
	n	%	n	%	n	%	n	%	n	%	n	%	N	%	χ^2^; *p*
Educational level															
1. tertiary	481	23.86	450	38.10	373	31.01	205	31.93	323	64.86	169	63.53	2001	34.46	856.21; 0.0001
2. secondary	835	41.42	186	15.75	574	47.71	305	47.51	162	32.53	87	32.71	2149	37.01	
3. vocational	700	34.72	545	46.15	256	21.28	132	20.56	13	2.61	10	3.76	1656	28.53	
Alcohol drinking															
1. non-drinkers	851	42.21	216	18.29	150	12.47	48	7.48	41	8.23	30	11.28	1336	23.01	1808.02; 0.0001
2. occasionally	1028	50.99	257	21.76	614	51.04	477	74.30	391	78.51	195	73.31	2962	51.02	
3. frequent drinkers	137	6.80	708	59.95	439	36.49	117	18.22	66	13.25	41	15.41	1508	25.97	
Cigarettes smoking															
1. never	1092	54.17	632	53.51	414	34.41	322	50.16	340	68.27	146	54.89	2946	50.74	354.65; 0.0001
2. quitted smoking	229	11.36	63	5.33	218	18.12	135	21.03	65	13.05	63	23.68	773	13.31	
3. smoker	695	34.47	486	41.16	571	47.47	185	28.82	93	18.67	57	21.43	2087	35.95	
Number of cigarettes smoked															
1. less than 5 cig. per day	351	50.50	79	16.26	86	15.06	36	19.46	40	43.01	9	15.79	601	28.80	285.96; 0.0013
2. 5–20 cig. per day	245	35.25	260	53.50	351	61.47	100	54.05	33	35.48	39	68.42	1028	49.26	
3. more than 20 cig. per day	99	14.25	147	30.24	134	23.47	49	26.49	20	21.51	9	15.79	458	21.94	
Coffee drinking															
1. no	961	47.67	152	12.87	35	2.91	123	19.16	158	31.73	30	11.27	1459	25.13	2439.36; 0.0001
2. 1–2 cups per day	937	46.48	337	28.54	247	20.53	298	46.42	300	60.24	188	70.68	2307	39.73	
3. more	118	5.85	692	58.59	921	76.56	221	34.42	40	8.03	48	18.05	2040	35.14	
Physical activity															
1. regularly	469	23.26	113	9.57	565	46.97	276	42.99	346	69.48	113	42.48	1882	32.41	1681.55; 0.0001
2. irregularly	1229	60.96	334	28.28	255	21.20	163	25.39	125	25.10	109	40.98	2215	38.15	
3. physically inactive	318	15.77	734	62.15	383	31.84	203	31.62	27	5.42	44	16.54	1709	29.44	

Legend: 1: 1986, 2: 1991, …, 6: 2021—number of survey; n—sample size with specific parameters; N—total sample size; %—percentage; χ^2^—chi-square test, *p*—*p*-value, level of statistical significance; cig.—cigarettes.

**Table 2:** Characteristics of women’s lifestyle in educational level and in particular years (N, percentages—%), the importance of the differences between years within educational level (Chi-square test, *p*—the level of significance) and the importance of the differences between educational level (Chi-square test, *p*—the level of significance).

	1: 1986		2: 1991		3: 1996		4: 2006		5: 2019		6: 2021		1–6: 1986–2021		
	n = 2016		n = 1181		n = 1203		n = 642		n = 498		n = 266		N = 5608		
	n	%	n	%	n	%	n	%	n	%	n	%	N	%	χ^2^; *p*
Alcohol drinking	1: Tertiary														358.47; 0.0001
1. no	166	34.51	28	6.22	44	11.80	9	4.39	25	7.74	19	11.24	291	14.54	
2. occasionally	271	56.34	63	14.00	173	46.38	161	78.54	254	78.64	122	72.19	1044	52.17	
3. frequent drinkers	44	9.15	359	79.78	156	41.82	35	17.07	44	13.62	28	16.57	666	33.28	
χ^2^; *p*	833.44; 0.0001														
	2: Secondary														
1. no	324	38.80	68	36.56	71	12.37	20	6.56	13	8.02	10	11.49	506	23.55	
2. occasionally	453	54.25	100	53.76	317	55.23	228	74.75	128	79.01	65	74.71	1291	60.07	
3. frequent drinkers	58	6.95	18	9.68	186	32.40	57	18.69	21	12.96	12	13.79	352	16.38	
χ^2^; *p*	365.35; 0.0001														
	3: Vocational														
1. no	361	51.57	120	22.02	35	13.67	19	14.39	3	23.08	1	10.00	539	32.55	
2. occasionally	304	43.43	94	17.25	124	48.44	88	66.67	9	69.23	8	80.00	627	37.86	
3. frequent drinkers	35	5.00	331	60.73	97	37.89	25	18.94	1	7.69	1	10.00	490	29.59	
χ^2^; *p*	633.79; 0.0001														
Cigarettes smoking	1: Tertiary														110.34; 0.0001
1. never	222	46.15	277	61.56	134	35.92	127	61.95	215	66.56	81	47.93	1056	52.77	
2. quitted smoking	87	18.09	15	3.33	79	21.18	38	18.54	52	16.10	47	27.81	318	15.89	
3. smoker	172	35.76	158	35.11	160	42.90	40	19.51	56	17.34	41	24.26	627	31.33	
χ^2^; *p*	191.07; 0.0001														
	2: Secondary														
1. never	422	50.54	86	46.24	217	37.80	149	48.85	119	73.46	59	67.82	1052	48.95	
2. quitted smoking	108	12.93	25	13.44	113	19.69	65	21.31	11	6.79	16	18.39	338	15.73	
3. smoker	305	36.53	75	40.32	244	42.51	91	29.84	32	19.75	12	13.79	759	35.32	
χ^2^; *p*	106.37; 0.0001														
	3: Vocational														
1. never	448	64.00	269	49.36	63	24.61	46	34.	6	46.15	6	60	838	50.60	
2. quitted smoking	34	4.86	23	4.22	26	10.16	32	24.24	2	15.38	0	0	117	7.07	
3. smoker	218	31.14	253	46.42	167	65.23	54	40.91	5	38.46	4	40	701	42.33	
χ^2^; *p*	176.71; 0.0001														
Number of cigarettes smoked	1: Tertiary														17.61; 0.0015
1. less than 5 cig. per day	72	41.86	26	16.46	27	16.88	7	17.50	22	39.29	4	9.76	158	25.20	
2. 5–20 cig. per day	63	36.63	108	68.35	82	51.25	21	52.50	23	41.07	31	75.61	328	52.31	
3. more than 20 cig. per day	37	21.51	24	15.19	51	31.88	12	30.00	11	19.64	6	14.63	141	22.49	
χ^2^; *p*	69.72; 0.0001														
	2: Secondary														
1. less than 5 cig. per day	164	53.77	10	13.33	43	17.62	22	24.18	15	46.88	5	41.67	259	34.12	
2. 5–20 cig. per day	97	31.80	9	12.00	169	69.26	48	52.75	9	28.13	5	41.67	337	44.40	
3. more than 20 cig. per day	44	14.43	56	74.67	32	13.11	21	23.08	8	25.00	2	16.67	163	21.48	
χ^2^; *p*	217.00; 0.0001														
	3: Vocational														
1. less than 5 cig. per day	115	52.75	43	17.00	16	9.58	7	12.96	3	60.00	0	0.00	184	26.25	
2. 5–20 cig. per day	85	38.99	143	56.52	100	59.88	31	57.41	1	20.00	3	75.00	363	51.78	
3. more than 20 cig. per day	18	8.26	67	26.48	51	30.54	16	29.63	1	20.00	1	25.00	154	21.97	
χ^2^; *p*	131.63; 0.0001														
Coffee drinking	1: Tertiary														144.14; 0.0001
1. no	204	42.41	36	8.00	5	1.34	45	21.95	104	32.20	17	10.06	411	20.54	
2. 1–2 cups per day	222	46.15	63	14.00	57	15.28	92	44.88	197	60.99	119	70.41	750	37.48	
3. more	55	11.43	351	78.00	311	83.38	68	33.17	22	6.81	33	19.53	840	41.98	
χ^2^; *p*	1073.07; 0.0001														
	2: Secondary														
1. no	365	43.71	47	25.27	25	4.36	55	18.03	49	30.25	11	12.64	552	25.69	
2. 1–2 cups per day	421	50.42	124	66.67	151	26.31	150	49.18	97	59.88	63	72.41	1006	46.81	
3. more	49	5.87	15	8.06	398	69.34	100	32.79	16	9.88	13	14.94	591	27.50	
χ^2^; *p*	887.47; 0.0001														
	3: Vocational														
1. no	392	56.00	69	12.66	5	1.95	23	17.42	5	38.46	2	20.00	496	29.95	
2. 1–2 cups per day	294	42.00	150	27.52	39	15.23	56	42.42	6	46.15	6	60.00	551	33.27	
3. more	14	2.00	326	59.82	212	82.81	53	40.15	2	15.38	2	20.00	609	36.77	
χ^2^; *p*	960.97; 0.0001														
Physical activity	1: Tertiary														213.00; 0.0001
1. regularly	136	28.27	34	7.56	146	39.14	81	39.51	217	67.18	60	35.50	674	33.68	
2. irregularly	269	55.93	56	12.44	85	22.79	57	27.80	86	26.63	76	44.97	629	31.43	
3. physically inactive	76	15.80	360	80.00	142	38.07	67	32.68	20	6.19	33	19.53	698	34.88	
χ^2^; *p*	775.70; 0.0001														
	2: Secondary														
1. regularly	188	22.51	43	23.12	279	48.61	133	43.61	121	74.69	50	57.47	814	37.88	
2. irregularly	507	60.72	120	64.52	143	24.91	76	24.92	34	20.99	29	33.33	909	42.30	
3. physically inactive	140	16.77	23	12.37	152	26.48	96	31.48	7	4.32	8	9.20	426	19.82	
χ^2^; *p*	393.72; 0.0001														
	3: Vocational														
1. regularly	145	20.71	36	6.61	140	54.69	62	46.97	8	61.54	3	30.00	394	23.79	
2. irregularly	453	64.71	158	28.99	27	10.55	30	22.73	5	38.46	4	40.00	677	40.88	
3. physically inactive	102	14.57	351	64.40	89	34.77	40	30.30	0	0.00	3	30.00	585	35.33	
χ^2^; *p*	624.71; 0.0001														

Legend: 1: 1986, 2: 1991, …, 6: 2021—number of survey; n—sample size with specific parameters; N—total sample size; %—percentage; χ^2^—chi-square test, *p*—*p*-value, level of statistical significance; cig.—cigarettes.

**Table 3:** Logistic regression analysis results for risk of alcohol consumption weekly or more, risk of current smoking cigarettes, risk of smoking more than 20 cigarettes a day, risk of drinking more than two cups a day, risk of being physically inactive for cohorts and level of education (OR, 95%CI, *p*) and χ^2^, R-square and Hosmer-Lemeshow tests results.

	OR	95%CI	*p*
Alkohol drinking—risk of alcohol consumption weekly or more			
Cohorts:			
1: 1986—reference group	1.0 (ref.)		
2: 1991	18.3546	14.8600–22.6711	0.0001
3: 1996	8.1047	6.5537–10.0227	0.0001
4: 2006	3.0510	2.3340–3.9884	0.0001
5: 2019	1.7028	1.2364–2.3452	0.0011
6: 2021	2.0486	1.3968–3.0044	0.0002
Level of education:			
1: tertiary—reference group	1.0 (ref.)		
2: secondary	0.4380	0.3708–0.5174	0.0001
3: vocational	0.6694	0.5654–0.7925	0.0001
χ^2^; *p*	209.36; 0.0001		
R-square (McFadden)	0.8658		
Hosmer-Lemeshow test; *p*	79.1956; 0.0001		
Cigarettes smoking—risk of current smoking			
Cohorts:			
1: 1986—reference group	1.0 (ref.)		
2: 1991	1.3153	1.1308–1.5300	0.0004
3: 1996	1.7900	1.5450–2.0740	0.0001
4: 2006	0.8020	0.6600–0.9747	0.0266
5: 2019	0.4922	0.3836–0.6316	0.0001
6: 2021	0.5821	0.4264–0.7948	0.0007
Level of education:			
1: tertiary—reference group	1.0 (ref.)		
2: secondary	1.1146	0.9735–1.2761	0.1163
3: vocational	1.4056	1.2187–1.6212	0.0001
χ^2^; *p*	64.82; 0.0001		
R-square (McFadden)	0.7706		
Hosmer-Lemeshow test; *p*	27.3277; 0.0001		
Number of cigarettes smoked—risk of smoking more than 20 cigarettes a day			
Cohorts:			
1: 1986—reference group	1.0 (ref.)		
2: 1991	2.6970	2.0060–3.6261	0.0001
3: 1996	1.8437	1.3827–2.4584	0.0001
4: 2006	2.1607	1.4629–3.1913	0.0001
5: 2019	1.6151	0.9365–2.7854	0.0847
6: 2021	1.1131	0.5252–2.3589	0.7799
Level of education:			
1: tertiary—reference group	1.0 (ref.)		
2: secondary	1.0410	0.7978–1.3585	0.7672
3: vocational	0.9056	0.6919–1.1852	0.4700
χ^2^; *p*	125.10; 0.0001		
R-square (McFadden)	0.2858		
Hosmer-Lemeshow test; *p*	14.1071; 0.0070		
Coffee drinking—risk of drinking more than two cups of coffee a day			
Cohorts:			
1: 1986—reference group	1.0 (ref.)		
2: 1991	19.9733	16.0029–24.9287	<0.0001
3: 1996	60.3562	47.6247–76.4912	<0.0001
4: 2006	8.8313	6.8642–11.3622	<0.0001
5: 2019	1.0922	0.7458–1.5600	0.6505
6: 2021	2.8215	1.9422–4.0989	<0.0001
Level of education:			
1: tertiary—reference group	1.0 (ref.)		
2: secondary	0.3509	0.2939–0.4189	0.0001
3: vocational	0.6353	0.5322–0.7584	0.0001
χ^2^; *p*	231.50; 0.0001		
R-square (McFadden)	0.9153		
Hosmer-Lemeshow test; *p*	59.2979; 0.0001		
Physical activity—risk of being physically inactive			
Cohorts:			
1: 1986—reference group	1.0 (ref.)		
2: 1991	7.8062	6.5836–9.2558	0.0001
3: 1996	2.5361	2.1329–3.0156	0.0001
4: 2006	2.5006	2.0310–3.0788	0.0001
5: 2019	0.2566	0.1700–0.3874	0.0001
6: 2021	0.9002	0.6329–1.2804	0.5587
Level of education:			
1: tertiary—reference group	1.0 (ref.)		
2: secondary	0.4858	0.4163–0.5669	0.0001
3: vocational	0.7449	0.6376–0.8703	0.0002
χ^2^; *p*	207.01; 0.0001		
R-square (McFadden)	0.8344		
Hosmer-Lemeshow test; *p*	82.2937; 0.0001		

Legend: 1: 1986, 2: 1991, …, 6: 2021—number of survey; OR—Odds Ratio; 95%CI—95% Confidence Interval; χ^2^—chi-square test, *p*—*p*-value, level of statistical significance.

**Table 4:** Logistic regression analysis results for risk of alcohol consumption weekly or more, risk of current smoking cigarettes, risk of smoking more than 20 cigarettes a day, risk of drinking more than two cups a day, risk of being physically inactive for women’s socioeconomic status and cohorts (OR, 95%CI, *p*) and χ^2^ and Hosmer-Lemeshow tests results.

	OR	95%CI	*p*
Alkohol drinking—risk of alcohol consumption weekly or more			
Level of education	0.7856	0.7207–0.8564	0.0001
Gini coefficient	4.0660	3.3488–4.9368	0.0001
Gender Inequality Index	5.5864	2.8479–10.9580	0.0001
Women total employment (%)	0.0493	0.0349–0.0695	0.0001
Employed women being in managerial positions (%)	25.6732	16.7658–39.3129	0.0001
Women among scientists (%)	0.6321	0.5758–0.6938	0.0001
Cohorts	458,975.4929	110,282.9073–1,910,164.5772	0.0001
χ^2^; *p*	1527.61; 0.0001		
Hosmer-Lemeshow test; *p*	16.0403; 0.0248		
Cigarettes smoking—risk of current smoking			
Level of education	1.1955	1.1129–1.2841	0.0001
Gini coefficient	0.0005	0.0001–3.0283	0.0001
Gender Inequality Index	1.8913	1.4295–2.5023	0.0001
Women total employment (%)	0.2499	0.1874–0.3333	0.0001
Employed women being in managerial positions (%)	4.9038	3.4152–7.0413	0.0001
Women among scientists (%)	0.6862	0.6315–0.7457	0.0001
Cohorts	469.7444	143.7170–1535.3766	0.0001
χ^2^; *p*	2551.31; 0.0001		
Hosmer-Lemeshow test; *p*	61.4640; 0.0001		
Number of cigarettes smoked—risk of smoking more than 20 cigarettes a day			
Level of education	0.9122	0.7977–1.0432	0.1796
Gini coefficient	13.3168	3.4261–51.7606	0.0004
Gender Inequality Index	1.5828	0.0001–4.8496	0.0042
Women total employment (%)	1.5943	0.9724–2.6140	0.0645
Employed women being in managerial positions (%)	0.4962	0.2636–0.9341	0.0299
Women among scientists (%)	1.4014	1.2163–1.6148	0.0001
Cohorts	0.0325	0.0042–0.2502	0.0010
χ^2^; *p*	1008.54; 0.0001		
Hosmer-Lemeshow test; *p*	107.8676; 0.0001		
Coffee drinking—risk of drinking more than two cups of coffee a day			
Level of education	0.7744	0.7101–0.8446	0.0001
Gini coefficient	1.0493	1.0016–5.9147	0.0001
Gender Inequality Index	7.4594	5.9971–9.2782	0.0001
Women total employment (%)	0.0170	0.0121–0.0237	0.0001
Employed women being in managerial positions (%)	80.3298	52.9453–121.8784	0.0001
Women among scientists (%)	0.4282	0.3921–0.4676	0.0001
Cohorts	69,525,170.9074	17,936,592.9111–269,490,900.8378	0.0001
χ^2^; *p*	1779.74; 0.0001		
Hosmer-Lemeshow test; *p*	67.9660; 0.0001		
Physical activity—risk of being physically inactive			
Level of education	0.8404	0.7760–0.9101	0.0001
Gini coefficient	5.1286	1.1096–23.7045	0.0001
Gender Inequality Index	3.6662	2.5864–5.1968	0.0001
Women total employment (%)	0.4572	0.3344–0.6250	0.0001
Employed women being in managerial positions (%)	1.6088	1.0774–2.4023	0.0201
Women among scientists (%)	1.0278	0.9447–1.1181	0.5244
Cohorts	104.97106	30.6414–359.6095	0.0001
χ^2^; *p*	1526.78; 0.0001		
Hosmer-Lemeshow test; *p*	31.6769; 0.0001		

Legend: OR—Odds Ratio; 95%CI—95% Confidence Interval; χ^2^—chi-square test, *p*—*p*-value, level of statistical significance.

## Data Availability

All relevant data are within the manuscript. The selected data underlying the results represented in the study are available from the author. There are ethical/legal restrictions on sharing a raw data set, which includes many confidential data (sensitive patient information) (EU Regulation EU, 2016/679 of the European Parliament and of the Council of 27 April 2016 on the protection of natural persons with regard to the processing of personal data and on the free movement of such data, and repealing Directive 95/46/EC—General Data Protection Regulation, https://eur-lex.europa.eu/eli/reg/2016/679/oj, accessed on 1 January 2022). If necessary, access will be given.
